# Ultrastructural and immunohistochemical evaluation of hyperplastic soft tissues surrounding dental implants in fibular jaws

**DOI:** 10.1038/s41598-024-60474-z

**Published:** 2024-05-10

**Authors:** Kezia Rachellea Mustakim, Mi Young Eo, Mi Hyun Seo, Hyeong-Cheol Yang, Min-Keun Kim, Hoon Myoung, Soung Min Kim

**Affiliations:** 1 Department of Oral and Maxillofacial Surgery, Dental Research Institute, School of Dentistry, Seoul National University, 101 Daehak-ro, Jongno-gu, Seoul, 03080 Korea; 2 Department of Dental Biomaterials Science, Dental Research Institute, School of Dentistry, Seoul National University, Seoul, Korea; 3 Department of Oral and Maxillofacial Surgery, College of Dentistry, Gangneung-Wonju National University, Gangneung, Korea; 4 Oral and Maxillofacial Microvascular Reconstruction LAB, Brong Ahafo Regional Hospital, P.O.Box 27, Sunyani, Ghana

**Keywords:** Endoplasmic reticulum stress, Fibula free flap, Hyperplastic tissue, Implant, Unfolded protein response, Foreign body granulation, Medical research, Pathogenesis

## Abstract

In reconstructive surgery, complications post-fibula free flap (FFF) reconstruction, notably peri-implant hyperplasia, are significant yet understudied. This study analyzed peri-implant hyperplastic tissue surrounding FFF, alongside peri-implantitis and foreign body granulation (FBG) tissues from patients treated at the Department of Oral and Maxillofacial Surgery, Seoul National University Dental Hospital. Using light microscopy, pseudoepitheliomatous hyperplasia, anucleate and pyknotic prickle cells, and excessive collagen deposition were observed in FFF hyperplastic tissue. Ultrastructural analyses revealed abnormal structures, including hemidesmosome dilation, bacterial invasion, and endoplasmic reticulum (ER) swelling. In immunohistochemical analysis, unfolded protein-response markers ATF6, PERK, XBP1, inflammatory marker NFκB, necroptosis marker MLKL, apoptosis marker GADD153, autophagy marker LC3, epithelial–mesenchymal transition, and angiogenesis markers were expressed variably in hyperplastic tissue surrounding FFF implants, peri-implantitis, and FBG tissues. NFκB expression was higher in peri-implantitis and FBG tissues compared to hyperplastic tissue surrounding FFF implants. PERK expression exceeded XBP1 significantly in FFF hyperplastic tissue, while expression levels of PERK, XBP1, and ATF6 were not significantly different in peri-implantitis and FBG tissues. These findings provide valuable insights into the interconnected roles of ER stress, necroptosis, apoptosis, and angiogenesis in the pathogenesis of oral pathologies, offering a foundation for innovative strategies in dental implant rehabilitation management and prevention.

## Introduction

Patients with malignancy, extensive benign conditions, or severe osteonecrosis in jaws are often treated with jaw resection to prevent the spread or recurrence of the disease. Extensive resections of the mandible or midface invariably result in aesthetic and oral function impairments. To address this challenge, osteocutaneous free flaps such as fibula free flap (FFF), have been employed for the reconstruction of complex facial areas^1^. The FFF has emerged as a cornerstone in the field of oral and maxillofacial surgery, offering a sophisticated solution for the intricate reconstruction of complex defects in the maxillofacial region. This innovative surgical technique involves the transplantation of a segment of the fibula, including its vascularized bone, skin, and potentially muscle components, to address challenging defects resulting from trauma, oncologic resections, or congenital abnormalities^2^. The FFF offers an abundant supply of bicortical bone for reconstructing defects across the midline and the ability to provide vascularized bone, making it a valuable contributor to oral rehabilitation, particularly in facilitating dental implant placement^3^. Nevertheless, a limitation of FFF is the bulkiness of its skin paddle, which may not be ideal for dental prosthetics^4^.

Current understanding emphasizes the importance of a narrow band of well-keratinized attached gingiva surrounding the implant for optimal peri-implant soft tissue health and implant longevity. However, when implants are placed through the skin, they are encompassed by a substantial layer of mobile tissue, potentially making them susceptible to peri-implantitis, which is a hyperplastic inflammatory response of the skin and subcutaneous tissue around implant abutments, leading to the formation of granulomatous tissue^5,6^. This condition may result in discomfort and bleeding during oral hygiene practices. A notable concern is the absence of specific data addressing this phenomenon, emphasizing the need for further investigation in this area.

Clinically, this hyperplasia tissue resembles fibrotic or scar tissue. To our knowledge, fibrosis is a pathological process characterized by the excessive deposition of extracellular matrix components, predominantly collagen, leading to tissue scarring and impaired organ function in response to chronic inflammation, injury, or abnormal wound healing^7^. Several articles have mentioned this growth and recurrence, but a clear microscopic structure has yet to be described^8–10^.

The response of the host following biomaterial implantation involves a series of sequential stages, encompassing injury, interactions with blood, provisional matrix formation, acute and chronic inflammation, granulation tissue development, foreign body reaction, and fibrosis/fibrous capsule formation^11^. Biomaterials are categorized based on their biocompatibility as either bioactive, biodegradable, bioinert, and/or biotolerant. Biotolerant materials are recognized by the host but remain segregated from host tissues via the development of a fibrous tissue layer (scar tissue). This fibrous tissue results from a minimal fibrotic response induced by the release of ions, corrosion products, and chemical compounds from the implant. The majority of synthetic polymers and metals falls within this biotolerant category^12^.

A study investigating the soft tissue reaction of muco-periosteal flaps to submerged titanium dental implants revealed a moderate but tolerable level of inflammation, leading to fibrosis^13^. Such persistent tolerance is attributable to the presence of healthy attached gingiva, which acts as a soft tissue seal, mitigating the risk of bacterial invasion and subsequent inflammation. The integrity of the soft-tissue seal is principally compromised through two mechanisms, i.e., instability of the peri-implant mucosa or that of the implant–abutment assembly^14^. However, in the context of the FFF, as previously mentioned, the absence of attached gingiva and the mobility of the skin facilitate chronic inflammation, precipitating excessive fibrosis and the development of hyperplastic tissue to fully encapsulate the implant. The current understanding considers well-established macrophages and fibroblasts to be the key players in biomaterial-mediated fibrosis^15^. In the present study, the author attempted to delve deeper into the cellular organelles that may play a role in the formation of peri-implant hyperplastic tissue, with a primary focus on the endoplasmic reticulum (ER).

ER stress (ERS) is a state where there is a buildup of unfolded or misfolded proteins in the ER lumen due to increased protein secretion or impaired ER protein folding that may trigger the unfolded protein response (UPR)^16^. Importantly, activated UPR pathways can initiate intercellular inflammatory signals, potentially contributing to the development of various diseases, including degenerative and fibrotic disorders in multiple organs^17,18^ which is described in Supplementary Fig. S1. Recent findings suggest that ERS and UPR signaling play crucial roles in various profibrotic mechanisms within distinct cell types^19^. In epithelial cells, ERS has been associated with apoptosis, inflammatory signaling, and initiation of the epithelial–mesenchymal transition. Conversely, in other cell types, ERS is correlated with myofibroblast activation, macrophage polarization, and differentiation of T-cells^18,20^.

Several diseases are correlated with ERS, including pulmonary fibrosis^21^, non-alcoholic fatty liver disease^22^, alcoholic liver disease^23^, chronic kidney disease^24^, cardiac fibrosis associated with heart failure^25^, Crohn’s disease^26^, liver cirrhosis^27^, hypertrophic scars and keloids^28,29^, chronic pancreatitis^30^, and neurofibromatosis^31^. In the author’s previous literature review, ERS was found to be correlated with the development of several oral diseases, including periodontitis, medication-induced gingival hyperplasia, salivary gland disease and malignancy, and oral squamous cell carcinoma^32^. To the author’s knowledge, the cellular structures of peri-implant hyperplastic tissue in fibula jaws have not been described. Building upon the author’s previous review article on the role of ERS in oral diseases^32^, the present study delved into the intricate mechanisms within this organelle to uncover a possible pivotal role of ERS in the development of the peri-implant hyperplastic tissue. This will contribute to a deeper understanding of the underlying pathology.

By conducting ultrastructural and immunohistochemical (IHC) analyses, this study seeks to unravel the intricate relationship between ERS and peri-implant hyperplastic tissue in the FFF. Through a comprehensive investigation, the author aimed to shed light on the underlying mechanisms and to gain basic insights into intraoral fibrosis, potentially opening new avenues for effective management and prevention of this recurrent challenge in dental implant rehabilitation.

The null hypothesis of the study posits that (1) there is no distinctive feature in peri-implant hyperplastic tissue in the FFF compared to peri-implantitis and foreign body granulation (FBG) tissues, and (2) ERS does not play a significant role in the development of peri-implant hyperplastic tissue in FFF-reconstructed jaws.

## Materials and methods

### Representative case presentation (Patient 1)

**Figure 1:**
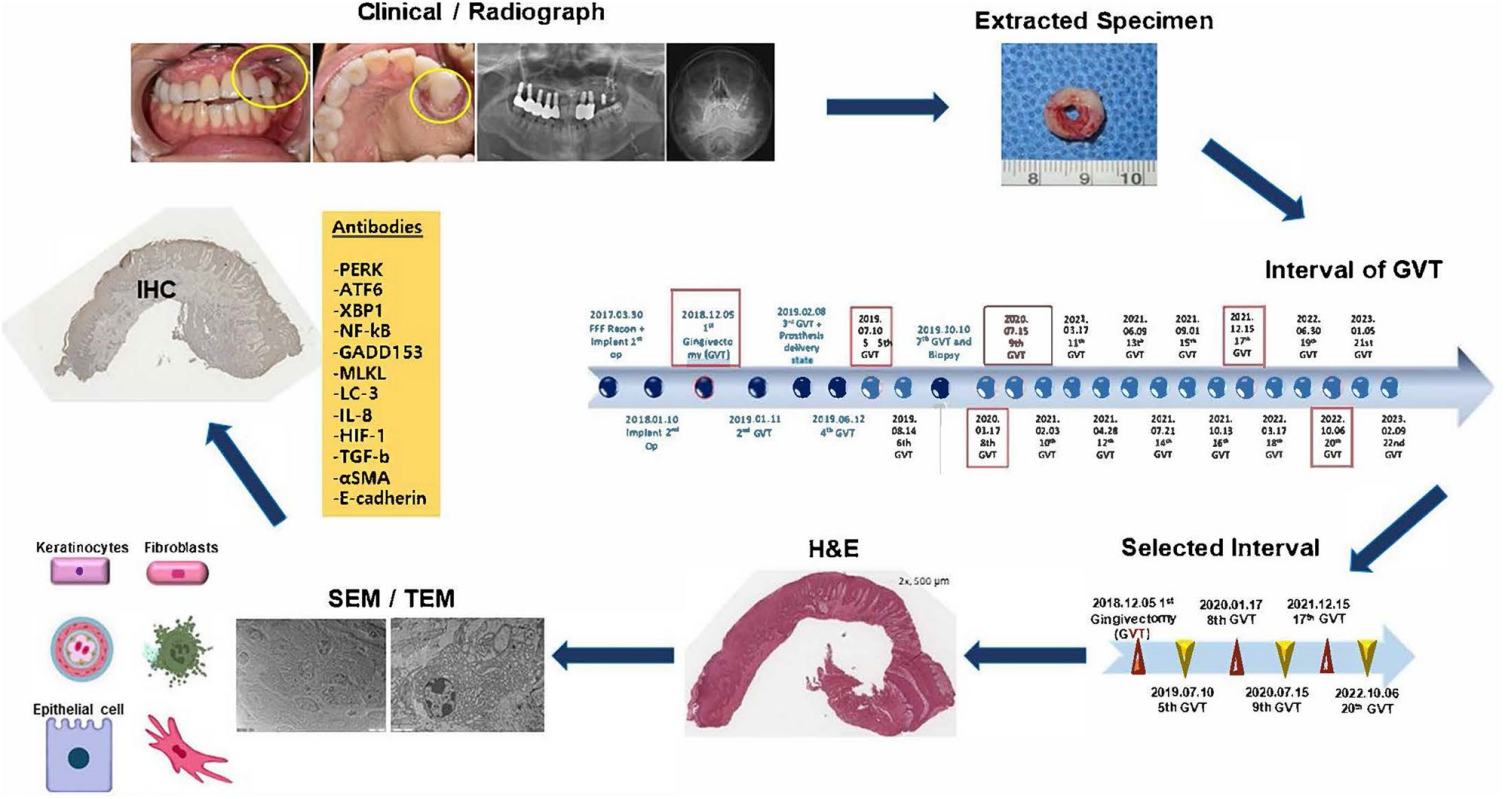
The flow of the current study: specimen collection and selection and preparation for histopathological, ultrastructural, and immunohistochemistry (IHC) analyses. Abbreviations: GVT, gingivectomy; H&E, hematoxylin–eosin; SEM, scanning electron microscope; TEM, transmission electron microscope. Antibodies abbreviations: *αSMA, α-smooth muscle actin; ATF6, activating transcription factor 6; E-cadherin, epithelial cadherin; GADD153, growth arrest-and DNA damage-inducible gene 153; HIF-1, hypoxia-inducible factor 1; IL-8, interleukin 8; LC3, microtubule-associated protein 1 light chain 3; MLKL. mixed lineage kinase domain-like protein; NFκB, nuclear factor kappa-light-chain-enhance of activated B cells; PERK, protein kinase RNA activated (PKR-like ER kinase); TGFβ, transforming growth factor beta; XBP1, xbox-binding protein 1.

A 20-year-old male patient was referred for reconstruction of the left maxillary defect due to a partial maxillectomy performed 11 years prior for fibroblastoma. In the radiogram, a malpositioned peg-shaped lateral incisor was observed in the left maxilla. Extraction of #22 and fistulectomy on the buccal side of #16 were performed. Prior to microvascular reconstruction, the initial treatment consisted of one year of obturator prosthesis delivery to temporarily separate the oral cavity from the nasal cavity, restoring the facial contour; improving mastication, articulation, and speech; and reducing drooling by providing buccal musculature and lip support. Computed tomography angiography was performed on both legs to check for vascularization and viability of the FFF; which was ultimately harvested from the left leg and transferred to the left maxilla. Following anastomosis, bone-level sandblasted, large-grit, acid-etched 4.1- × 8.0-mm Straumann Regular CrossFit^®^ (Institut Straumann AG, Basel, Switzerland) implant fixtures were installed in #22, #23, and #26. Following reconstruction, the patient was followed closely every month with endoscopic-assisted sinus irrigation to check the oro-nasal lining condition. At 20 months of follow-up after reconstruction and implant installation, a re-entry procedure using a 6.0-mm healing abutment Straumann for #22 and #23 and defatting were carried out simultaneously. One month following the re-entry procedure, doughnut-shaped hyperplastic tissue covering the implant abutment was observed and removed; one month later, the hyperplastic tissue reappeared and was removed again. Eight months following the re-entry procedure, the definitive prosthesis was delivered together with hyperplastic tissue removal. The patient was clinically and radiographically followed periodically at 1 month, 3 months, 4 months, and 6 months to check the implant and flap status. At each follow-up, the hyperplastic tissue was observed to have regrown after removal, and routine removal was required. However, no radiographical marginal bone loss or clinical mobility was observed during routine follow-up. The specimens were then collected and selected for further analyses (Fig. 1).

### Specimen selection of hyperplastic tissue surrounding the implant, peri-implantitis tissue, and FBG tissue

We analyzed hyperplastic tissue surrounding implants as well as peri-implantitis tissue and FBG tissue retrieved from the Department of Oral Maxillofacial Surgery at Seoul National University School of Dentistry. This study, along with its access to patient records for research purposes, was ethically approved by the Seoul National University Institutional Review Board (S-D20220024). The hyperplastic tissue surrounding implant specimens was obtained from patients who underwent implant rehabilitation following FFF microvascular reconstruction surgery in the jaw. All patients were selected from among those treated by a single surgeon during 2018–2023. During routine follow-up, these patients received check-ups and treatment for implant maintenance. The specimens were retrieved by excision using a scalpel and stored in normal saline, 10% buffered formalin, or 2.5% glutaraldehyde.

To identify distinctive characteristics for comparison, peri-implantitis and FBG tissues were selected from our laboratory specimen bank. These specimens were collected as part of routine laboratory work for further analyses and were kept in normal saline, 10% formalin, or 2.5% glutaraldehyde. The rationale for selecting peri-implantitis and FBG tissues growing onto reconstruction plates is that the origin of these tissues is the oral mucosa.

Peri-implantitis tissues were obtained from patients who underwent peri-implantitis treatment, including implant removal or curettage. Granulation tissue was obtained from the FBG that surrounded the exposed mandible reconstruction plate.

All specimens underwent a screening process. For hyperplastic tissue surrounding implants in FFF-reconstructed jaws, specimens that exhibited a “doughnut” were selected.

For the peri-implantitis groups, specimens were chosen based on patient data. Peri-implant tissues that were excised due to malignancy surrounding implants, sinusitis, or osteomyelitis were excluded from the groups. The specimens were categorized as follows:

1. Comparative group: contained tissues of oral mucosal origin; peri-implantitis tissues (C-1, C-2, C-3) and FBG tissue (C-4) (n = 4)
2. Experimental group: contained hyperplastic tissues surrounding implants in FFF-reconstructed jaws (n = 14); these tissues were categorized based on the tissue-excision interval (A, first occurrence; B, recurrence at 1 month; Ce, recurrence at 2 months; D, recurrence at 3 months; E, recurrence at 4 months; F, recurrence at 6 months; G, recurrence at 1 year; H, recurrence at ≥ 1 year)

### Specimen preparation for histopathological analysis

The tissue sections were prepared for examination under a light microscope by dehydration, clearing, and impregnation. Dehydration was achieved using ethanol, starting from a concentration of 70% and progressing to a concentration of 100%. The dehydrated tissues were cleared using Neo-clear^®^ (Aruimea, Madrid, Spain) and then embedded in paraffin according to the following sequence: Neo-clear^®^ 100% → Neo-clear^®^ 100% → Neo-clear^®^ 100% → paraffin 100% → paraffin 100%. The paraffin-embedded tissue blocks were cut into serial sections measuring 4 µm thick and then stained with hematoxylin and eosin. The resulting slides were scanned using a digital pathology slide scanner (Aperio CS2^®^; Leica, Wetzlar, Germany) and examined using a digital slide viewer application (SlideViewer 2.6^®^; 3DHISTECH, Budapest, Hungary).

### Specimen preparation for scanning electron microscopy (SEM) and transmission electron microscopy (TEM)

The specimens were fixed in 2.5% glutaraldehyde following excision for ≥ 3 days. Subsequently, the specimens were dissected into 1 × 1 × 1-mm blocks, embedded in epoxy resin, and sliced into ultra-thin sections measuring 70–80 nm thick. To aid in the preliminary assessment of tissue morphology, sections with a thickness of 1 µm were stained with toluidine blue and examined using a BX41 light microscope (Olympus Co., Tokyo, Japan).

For more in-depth investigations, TEM was conducted using a JEM-1400 Flash^®^ microscope (Jeol Ltd., Tokyo, Japan) operated at 120 kV. Images were captured at various magnifications, including 1000×, 2000×, 3000×, 5000×, and 10,000×. SEM was performed using an Apreo S^®^ microscope (Thermo Fisher Scientific, Waltham, MA, USA) with an operating voltage of 5 kV. SEM images were acquired at magnifications of 250×, 500×, 1000×, 2500×, 5000×, 10,000×, and 30,000×.

The examination encompassed the entire tissue structure from the epidermis to the reticular dermis layer, enabling comprehensive exploration of the tissue microenvironment and identification of organelles contributing to the development of hyperplastic tissue surrounding the implant in the FFF, peri-implantitis tissue, and FBG tissue. In addition, the ER profile in fibroblasts and prickle cells was scrutinized.

### IHC analysis

For IHC staining, paraffin-embedded tissues were cut into 4-µm-thick sections and transferred onto microscope slides using a semi-automated rotary microtome (Leica Biosystems, Wetzlar, Germany). Each microscope slide was evaluated under the BX41 light microscope (Olympus Co., Tokyo, Japan) to confirm the presence of all tissue structures. Antibodies involved in the UPR, inflammatory signaling, programmed cell death, angiogenesis, or epithelial–mesenchymal transition were selected for IHC analysis, as shown in Table 1.

**Table 1:** Antibodies used in this study for immunohistochemistry analysis.

Pathways/signaling	Number	Antibodies
Unfolded protein response	3	PERK, ATF6, XBP1
Inflammatory related	1	NFκB
Programmed cell death		
Apoptosis	1	CHOP/GADD153
Necroptosis	1	MLKL
Autophagy	1	LC3
Angiogenesis	2	IL-8, HIF-1
Epithelial-mesenchymal transition	3	TGF-β, αSMA, E-cadherin
Total	12	

All antibodies were obtained from Santa Cruz Biotechnology, USA.

*αSMA, α-smooth muscle actin; ATF6, activating transcription factor 6; CHOP/GADD153, CCAAT-enhancer-binding protein homologous protein/growth arrest-and DNA damage-inducible gene 153; E-cadherin, epithelial cadherin; HIF-1, hypoxia-inducible factor 1; IL-8, interleukin 8; LC3, microtubule-associated protein 1 light chain 3; MLKL. Mixed lineage kinase domain-like protein; NFκB, nuclear factor kappa-light-chain-enhance of activated B cells; PERK, protein kinase RNA activated (PKR-like ER kinase); TGFβ, transforming growth factor beta; XBP1, xbox-binding protein 1.

In the IHC protocol, paraffin-embedded tissue sections underwent paraffin removal, which involved drying in the oven for 1 h, sequential immersion in xylene and ethanol with concentration reduction from 100 to 50%, and a final distilled water rinse. Antigen retrieval was achieved through Proteinase K treatment and washing with phosphate-buffered saline. Endogenous peroxidase activity was blocked with 3% H_2_O_2_ for 30 min to remove the blood cells and avoid false-positive results, followed by background blocking using 10% donkey serum. Primary antibodies were applied and incubated overnight at refrigerated temperatures. Slides were then left at room temperature for 2 h. Universal secondary antibodies were applied, followed by avidin–biotin complex reagent and diaminobenzidine (DAB) substrate application for 5 min, with monitoring for brown coloration. Hematoxylin staining was performed, followed by cover glass application.

For quantitative IHC analysis, we employed an open-source digital pathology software program (QuPath^®^; Northern Ireland Molecular Pathology Lab., Centre for Cancer Research and Cell Biology, Queen’s University, Belfast, UK) as a fundamental component of our materials and methods^33^. QuPath^®^ has proven to be an indispensable tool for research, providing a comprehensive platform for the accurate and efficient assessment of IHC staining patterns and biomarker expression within tissue samples^34–36^. Automated IHC measurements offer a solution to the challenges associated with subjective visual scoring by pathologists. Whole-slide imaging systems readily transform glass slides into high-quality digital images. These automated measurements demonstrate precision, especially in detecting subtle staining that may be difficult for the human eye to discern. Additionally, they generate continuous and reliable data. When integrated into the visual scoring process, computer-aided IHC analysis significantly enhances both intra- and inter-observer agreement among pathologists^37,38^.

The analysis process involved importing IHC images into QuPath^®^ and creating a structured project workspace. Within this workspace, we established representative brightfield H-DAB stain vectors encompassing the representative area consisting of representative hematoxylin and DAB stains and a background area set to auto. Subsequently, we defined regions of interest (ROIs) using QuPath^®^’s “create thresholder” annotation tool, enabling precise localization of target tissue regions. For angiogenesis using interleukin-8 and HIF antibodies, the reticulum dermis with abundant blood vessels was selected as an ROI using the rectangle annotation. To quantify staining characteristics, we used QuPath^®^’s positive cell-detection algorithms to automatically identify and classify cells within the ROIs, facilitating the calculation of quantitative data for each cell (Supplementary Table S2). The Histoscore (H-score) of the positive slide was generated and subjected to further statistical analysis (Supplementary Fig. S3). The H-score serves as a quantitative metric for the transformation of traditional IHC into a more objective range. It is determined considering both the intensity of staining and the proportion of stained cells within a given sample^37^. In our study, H-scores were interpreted as < 10 points (negative), 10–100 points (low), 101–200 points (moderate), and > 200 points (intense), as described in previous literature^39^.

### Statistical analysis

For positive cell-detection analysis by QuPath^®^, the H-scores were calculated. The data normal distribution was tested by the Shapiro–Wilk test. Analyses were performed for comparisons between the comparative and experimental groups and for comparisons within the experimental group. The difference between two groups was tested by Student’s *t* test, while, for more than two groups, analysis of variance with post-hoc Tukey’s test was applied. Statistical analysis was carried out using SPSS version 26.0^®^ (IBM Corp., Armonk, NY, USA). *P* < 0.05 was considered statistically significant.

### Ethical approval

The study protocol complied with the principles of the Declaration of Helsinki and was approved by the Seoul National University Institutional Review Board (S-D20220024). All methods were performed in accordance with the relevant guidelines and regulations. All patients were informed of the surgical procedure with the potential risks and benefits, and an informed consent was obtained to receive the treatment and to be included in the study.

## Results

### Patients and selected specimens

The comparative group comprised four specimens from three patients with peri-implantitis (labeled as C-1, C-2, and C-3), along with one FBG specimen (labeled as C-4) (Supplementary Table S4).

In experimental groups, 14 specimens were obtained from four patients. For ease of identification, the patients were labeled as Patient 1, Patient 2, Patient 3, and Patient 4 (Supplementary Table S5). Additionally, the specimens were labeled according to the tissue-excision interval, as follows (e.g., “A-2” indicates “recurrence tissue excised at 1 month in Patient 2”; Supplementary Table S6):

- A: First occurrence (n = 2; A-1, A-2)
- B: Recurrence, excision at one month (n = 1; B-1)
- Ce: Recurrence, excision at two months (n = 1; Ce-1)
- D: Recurrence, excision at three months (n = 3; D-1. D-2, D-3)
- E: Recurrence, excision at four months (n = 2; E-1, E-3)
- F: Recurrence, excision at six months (n = 3; F-1, F-3–1, F-3–2)
- G: Recurrence, excision at one year (n = 1; G-4)
- H: Recurrence, excision after more than one year (n = 1; H-4)

### Histopathology findings

1. Experimental group

**Figure 2:**
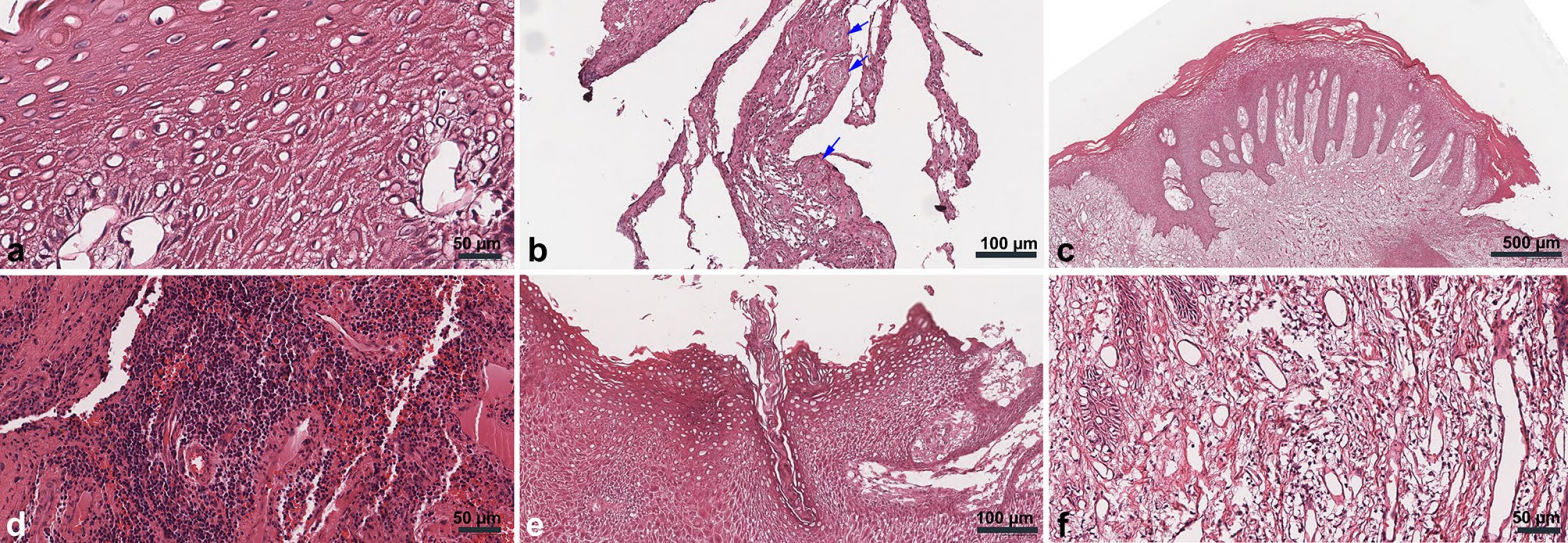
Histology findings from peri-implant hyperplastic tissue. The stratum spinosum in specimen A-2 was filled by pyknotic and anucleated prickle cells (**A**). Keratin granules were observed in the B-1 specimen (**B**; arrows). Pseudo-epitheliomatous hyperplasia of the epidermis on the Ce-1 slide (**C**). Abundant inflammatory cells on slide D-3 (**D**). Vellus hair without a functional structure was observed on slide F-1 (**E**). Collagen fibers and abundant blood vessels on slide H-4 (**F**).

The tissues exhibited a keratinized squamous epithelium with pseudoepitheliomatous hyperplasia features in the epidermal layer. Furthermore, anastomosing strands of the epidermal layer into the reticular dermis were observed. Acanthosis and dyskeratosis of the stratum spinosum, as well as keratin/epithelial pearls, were evident. Vellus hair in the epidermis layer without a functional structure, such as the arrector pili muscle or a sweat gland extending only until the middle portion of the stratum spinosum, was observed in F-1. In the reticular dermis, abundant inflammatory cells, small blood vessels including venules and arterioles, and fibroblasts, along with collagen disposition, were observed (Fig. 2).

(2) Comparative group

Peri-implantitis specimens consisted of fibrotic tissue rich in collagen fibers, small blood vessels, and inflammatory cells. In C-1, several pieces of bone tissue with empty osteocytes indicating sequestrum were observed. The epidermal layer consisted of nucleated prickle cells (Supplementary Fig. S7a–c). The C-4 consisted of thin keratinized stratified squamous epithelial with acanthosis of the stratum spinosum with nucleated prickle cells. The dermis layer consisted of fibrotic reticular dermis with abundant inflammatory cells and small blood vessels (Supplementary Fig. S7d).

### SEM and TEM findings

1. Experimental group

In the first occurrence, the A-1 and A-2 specimens showed anucleated prickle cells in the stratum spinosum. Neutrophils were the most abundant inflammatory cells, along with a significant number of monocytes. In the reticular dermis, the formation of blood vessels and thick connective tissue was observed (Supplementary Fig. S8a1–b3). In the Ce-1 specimen, the stratum spinosum was filled with mixed anucleated and nucleated prickle cells, indicating the differentiation and maturation of keratinocytes. The stratum basalis displayed an abnormal structure with dilated hemidesmosomes between cells. Stellate and spindle-shaped fibroblasts surrounding the thick connective tissue and endothelial cells were observed (Supplementary Fig. S8c1–d3).

**Figure 3:**
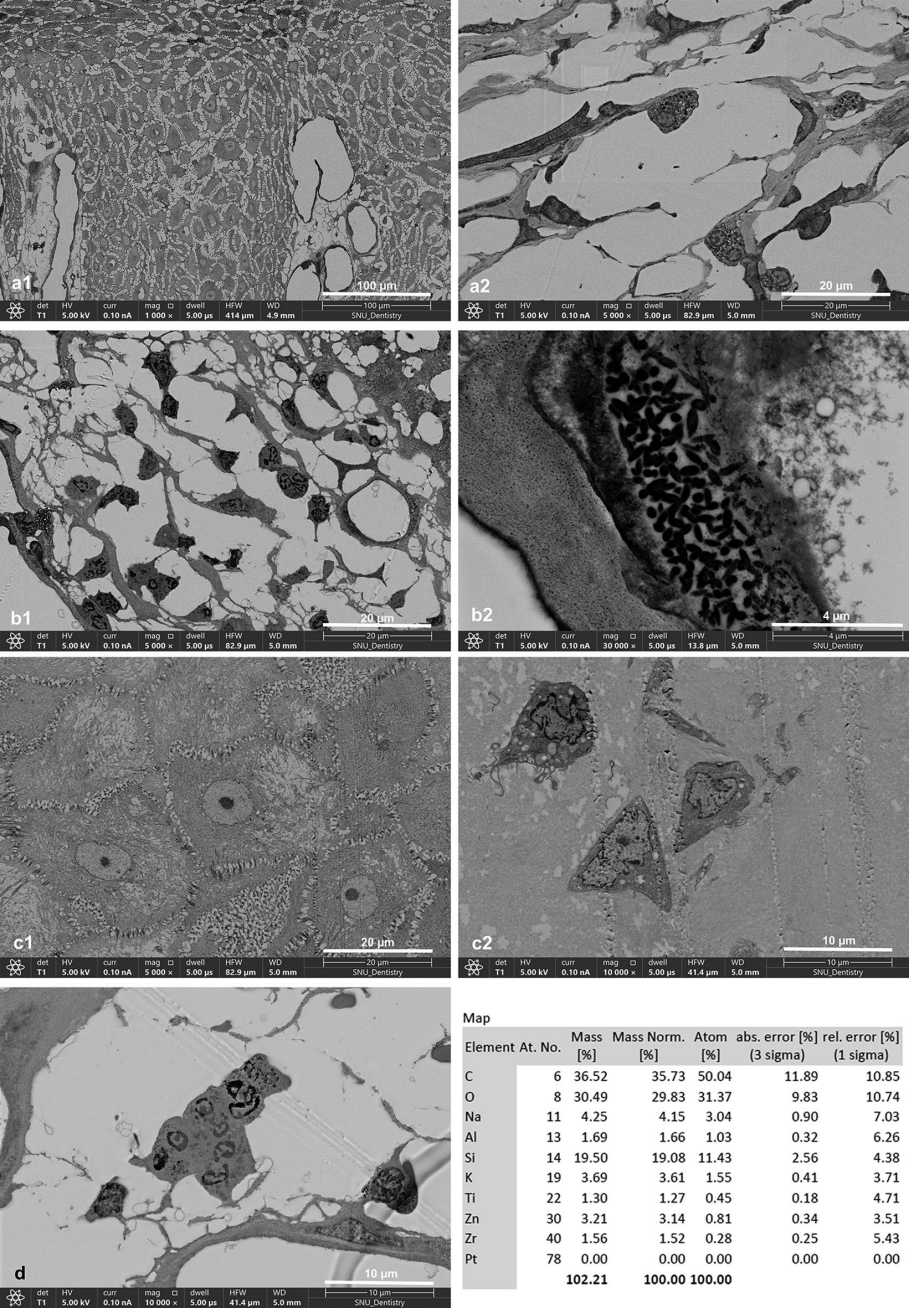
SEM findings from the D-1 specimen revealed stratum spinosum consisting of mixed anucleated and nucleated prickle cells (**a**1). Mast cells, eosinophils, and lymphocytes were observed in D-1 (**a**2). Abundant neutrophils were observed in the D-2 specimen (**b**1), with bacterial invasion in the cell (**b**2). The D-3 specimen showed metaplasia of prickle cells into cuboidal cells (**c**1), and proto-myofibroblasts were observed in the reticular dermis (**c**2). A multinucleated giant cell was observed in D-2, and the energy dispersive x-ray revealed components of titanium, aluminum, zirconium, and zinc (**d**).

Mast cells were observed in the D-1 specimen, and the D-2 specimen showed abundant neutrophils and bacterial invasion into the cells. In addition, a multinucleated giant cell was observed in the reticular dermis, and an energy dispersive x-ray showed components of titanium, aluminum, zinc, and zirconium. In the D-3 specimen, the epidermal layer exhibited the metaplasia of prickle cells into cuboidal cells, and proto-myofibroblasts were observed in the reticular dermis. Additionally, autophagocytosis was observed (Fig. 3). The E-1 specimen showed the process of angiogenesis and maturation of functional blood vessels containing various types of peripheral blood cells; it was characterized by the aggregation of endothelial cells to form a lumen and eventually the maturation of the vessels. These blood vessels were surrounded by inflammatory cells and fibroblast cells, which decreased in number as the blood vessels matured (Supplementary Fig. S9). The details of the peripheral blood vessels can be seen in Supplementary Fig. S10. Neutrophils, lymphocytes, macrophages, and plasma cells were dominant (Supplementary Fig. S11). The reticular dermis showed abundant stellate and spindle-shaped fibroblasts and collagen (Supplementary Fig. S9). Cell interactions were observed between mononuclear cells with apoptotic bodies, including both apoptosis and oncosis (Supplementary Fig. S11). Meanwhile, the E-3 specimen showed the metaplastic process by which prickle cells were transformed into cuboidal cells (Supplementary Fig. S12).

The F-3-1 specimen showed acanthosis with the metaplasia of prickle cells into cuboidal cells. The metaplastic cells exhibited lower electron density, making them appear brighter than the denser prickle cells. Within the denser prickle cells, cellular activity involving the migration of keratin granules was observed at the intercellular desmosomes. In the reticular dermis, various inflammatory cells, including macrophages, dendritic cells, plasma cells, and neutrophils, were observed alongside programmed cell death (Supplementary Fig. S12). Fibroblasts and proto-myofibroblasts were also present in the reticular dermis (Supplementary Fig. S12C1). The G-4 specimen showed metaplasia of the prickle cells in the epidermal layer, with abundant lymphocytes and dendritic cells. The dense prickle cells were observed from the anastomosing strand of the epidermal layer into the reticulum dermal layer (Supplementary Fig. S13). The H-4 specimen showed bacterial invasion in most cells (Supplementary Fig. S12C3–4).

(2) ER profile in the fibroblasts and prickle cells of each group

**Figure 4:**
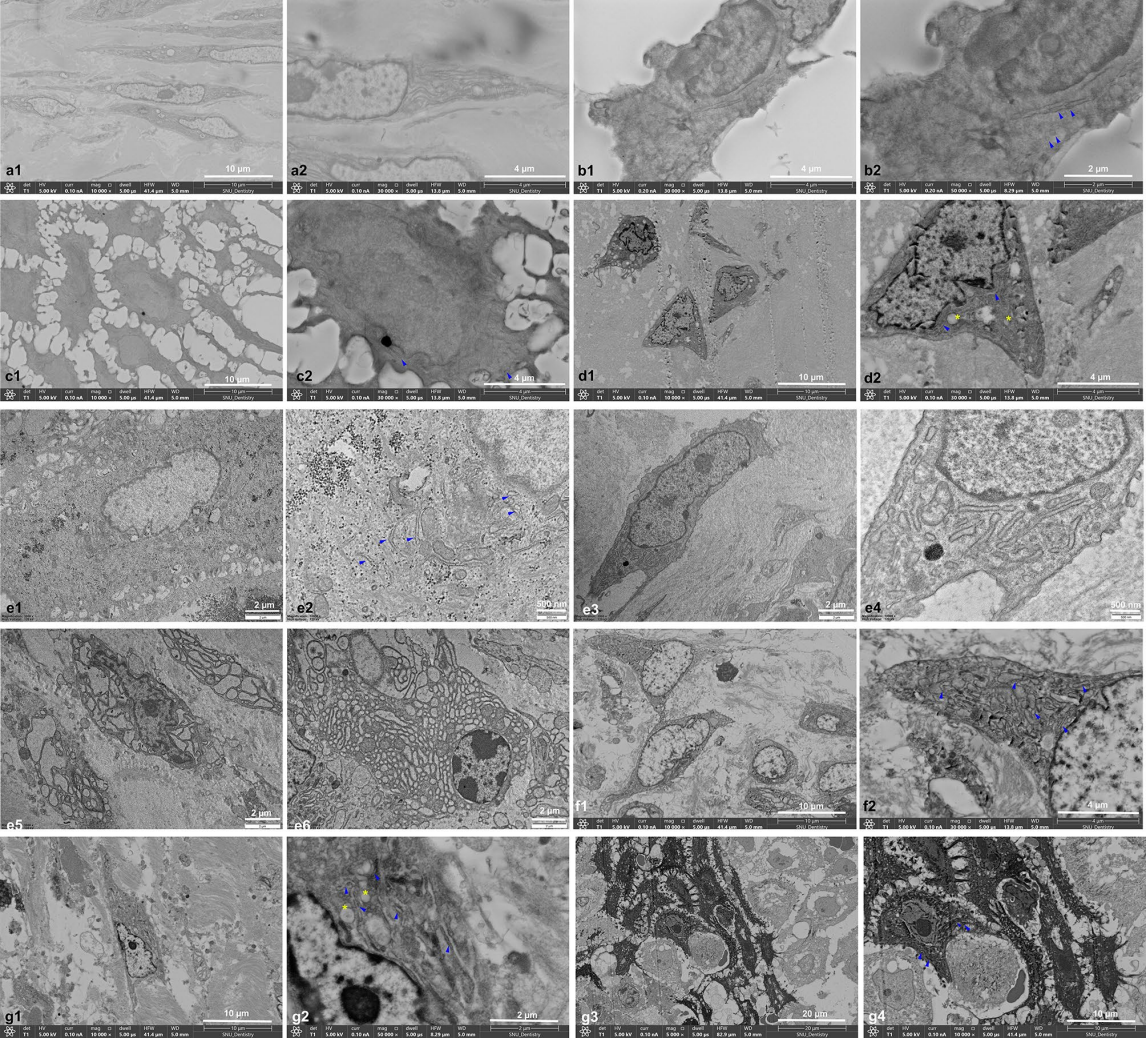
Fibroblast cells with early signs of ERS. The continuation of the ER can still be seen, although swelling and fragmentation of the ER are beginning to be visible (**a**1-2). Swelling, fragmentation, and vacuolization of the ER were observed in fibroblasts (**b**1-2; representative images of group A, **d**1-2; representative images of group D, **e**3–5; representative images of group E, **f**1-2; representative images of group F, **g**3-4; representative images of group G) and prickle cells (**c**1-2; representative images of group C, **e**1-2; representative images of group E, **g**1-2; representative images of group G) from each group A, C, D, E, F, G. In the TEM examination, severely dilated ER cisternae were observed (**d**5, **d**6).

Swelling, fragmentation, and vacuolization of the ER were observed in the fibroblasts and prickle cells of each specimen. In the TEM examination, an island of cells in the reticular dermis with an abnormal ER characterized by severely dilated cisternae was observed (Fig. 4).

(3) Comparative groups

The C-2 specimen exhibited an active inflammatory response and degenerative changes, characterized by the presence of lysosomes and swelling of the ER cisternae and mitochondria in most cells, especially the fibroblasts. Most of the inflammatory cells were dendritic cells, although plasma cells were also observed. On the other hand, C-1 and C-3 showed the characteristics of chronic inflammation, with most cells undergoing degenerative changes and necrosis and almost no inflammatory cells, except mast cells, observed. Furthermore, ER cisterna dilation was observed in the fibroblasts (Supplementary Fig. S14). On the other hand, in C-4, the epidermal layer showed compact and intact stratum basalis structures with intact hemidesmosomes between cells. Various inflammatory cells, including monocytes, macrophages, plasma cells, dendritic cells, and neutrophils, were observed. In the reticulum dermis, the ER showed swelling of the cisternae (Supplementary Fig. S15).

### IHC findings

- Comparison between the experimental and comparative groups

The H-scores from the IHC analysis are depicted in Tables 2 and 3.

**Table 2:** IHC expression among the groups.

Antibody	Experimental			Comparative			A.B *p*-value
	Min. H score	Max. H score	Mean ± SD (A)	Min. H score	Max. H score	Mean ± SD (B)	
ATF-6	31.46	150.25	80.07 ± 27.84	33.77	125.88	77.46 ± 38.89	0.881
PERK	54.47	117.41	92.16 ± 18.27	61.49	141.90	91.00 ± 9.81	0.935
XBP1	34.32	86.13	63.09 ± 14.91	30.02	105.58	65.41 ± 31.13	0.840
NFκB	57.54	145.87	126.33 ± 29.96	167.66	176.15	163.71 ± 12.94	0.029*
GADD153	76.99	199.47	106.48 ± 35.31	60.91	155.44	104.30 ± 47.82	0.921
MLKL	135.58	173.54	156.71 ± 12.82	128.37	192.058	158.32 ± 26.09	0.862
LC3	95.18	166.66	119.44 ± 23.71	84.48	162.32	124.90 ± 33.48	0.722
E-cadherin	29.94	125.18	78.12 ± 30.52	33.91	125.72	71.61 ± 38.79	0.734
TGF-β	4.95	90.12	44.23 ± 26.86	13.50	54.12	32.47 ± 17.98	0.429
αSMA	1.44	127.81	47.32 ± 41.45	13.80	111.51	64.00 ± 49.49	0.510
HIF	0.37	48.60	15.69 ± 20.49	7.53	19.50	13.52 ± 8.47	0.892
IL-8	1.73	25.23	8.03 ± 9.75	16.56	98.77	57.67 ± 58.13	0.083

H scores were calculated with Qupath^®^ (Northern Ireland Molecular Pathology Laboratory, Centre for Cancer Research and Cell Biology, Queen’s University Belfast, Belfast, United Kingdom). H score interpretation: < 10 (negative), 10–100 (low), 101–200 (moderate), > 200 (intense). Student-t test was performed for average score of H score with *p value < 0.05 was considered statistically significant.

All antibodies were obtained from Santa Cruz Biotechnology, USA. *αSMA, α-smooth muscle actin; ATF6, activating transcription factor 6; CHOP/GADD153, CCAAT-enhancer-binding protein homologous protein/growth arrest-and DNA damage-inducible gene 153; E-cadherin, epithelial cadherin; HIF-1, hypoxia-inducible factor 1; IL-8, interleukin 8; LC3, microtubule-associated protein 1 light chain 3; MLKL. Mixed lineage kinase domain-like protein; NFκB, nuclear factor kappa-light-chain-enhance of activated B cells; PERK, protein kinase RNA activated (PKR-like ER kinase); TGFβ, transforming growth factor beta; XBP1, xbox-binding protein 1.

**Table 3:** Unfolded protein response related antibodies H score comparison within experimental and comparative groups.

Group	Average H-score			A-B sig	A-C sig	B-C sig	Overall *p*-value
	PERK (A)	ATF6 (B)	XBP1 (C)				
Experimental	92.16 ± 18.27	80.07 ± 27.84	63.09 ± 14.91	0.338	0.006*	0.125	0.008*
Comparative	91.00 ± 9.81	77.46 ± 38.89	65.41 ± 31.13	0.859	0.595	0.886	0.622

Statistical analysis was performed using one-way ANOVA and post-hoc Tukey test with *p value < 0.05 was considered statistically significant. A-B sig.: Significance of comparison between PERK and ATF-6 H score expression; A-C sig.: Significance of comparison between PERK and XBP1 H score expression; B-C sig.: Significance of comparison between ATF-6 and XBP1 H score expression.

1. UPR expression

**Figure 5:**
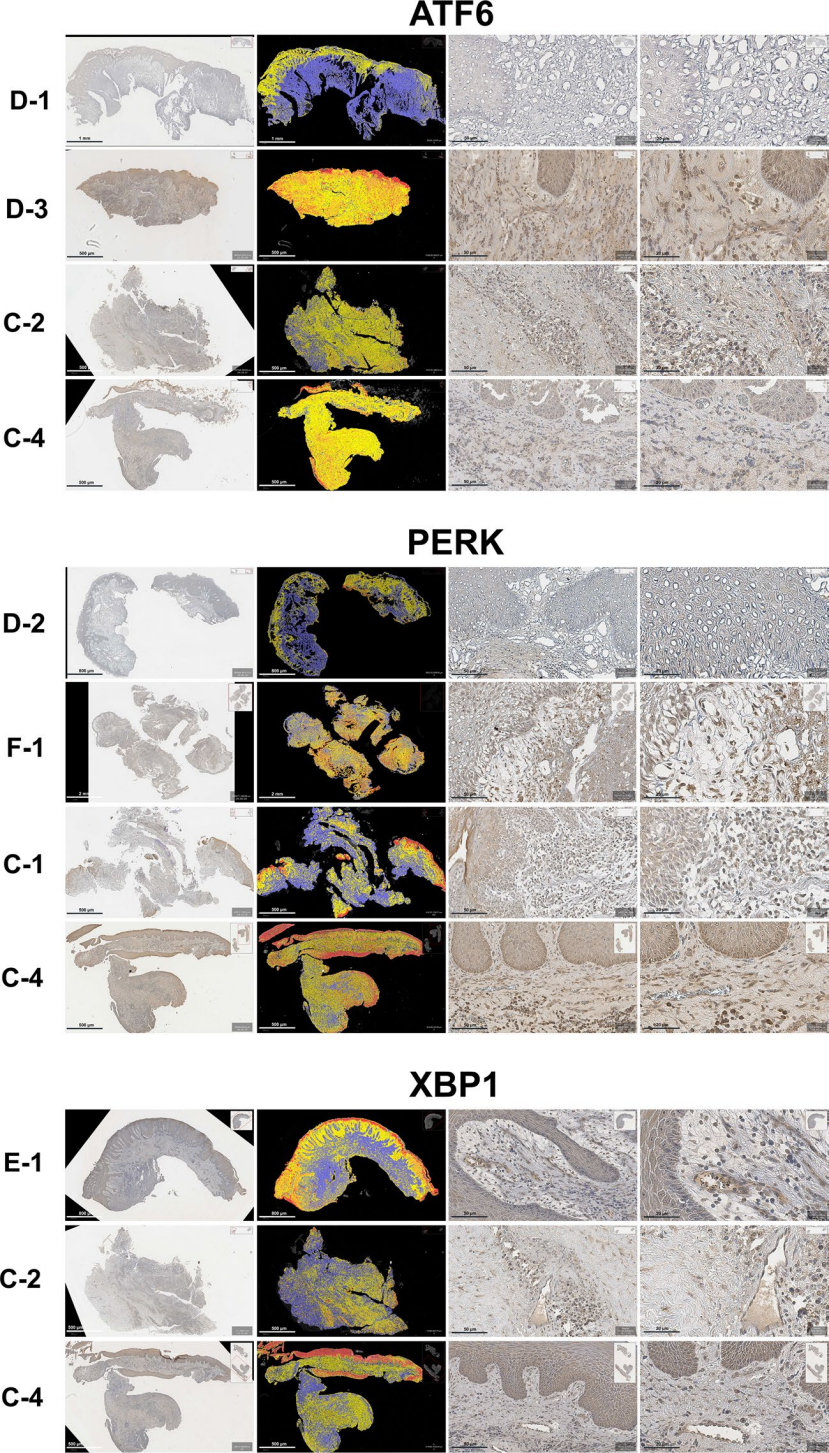
The IHC for UPR.

The UPR, marked by protein kinase RNA-like ER kinase (PERK), activating transcription factor 6 (ATF6), and X-box binding protein 1 (XBP1) expression, was observed in all groups. The expression for PERK ranged from low to moderate in both the experimental and comparative groups, with average H-scores of 92.16 ± 18.27 and 91.00 ± 9.81, respectively. The ATF6 level also ranged from low to moderate in both the experimental and comparative groups, with average H-scores of 80.07 ± 27.84 and 77.46 ± 38.89, respectively. XBP1 expression in the experimental group was low, and it was low to moderate in the comparative group, with average H-scores of 63.09 ± 14.91 and 65.41 ± 31.13, respectively. None of those H-scores differed significantly between the experimental and comparative groups (*p* > 0.05). Within the experimental group, the H-scores for the three proteins did differ significantly, with PERK having the highest H-score and XBP1 having the lowest one (*p* < 0.05) (Fig. 5).

(2) Inflammatory signaling

Inflammatory signaling, marked by the NF-κB antibody, was low to moderate in the experimental group, with an average H-score of 126.33 ± 29.96, whereas the comparative group showed moderate expression and a significantly higher H-score than the experimental group, 163.71 ± 12.94 (Supplementary Fig. S16).

(3) Apoptosis

Apoptosis was marked by the GADD153 antibody, and its expression ranged from low to moderate in both the experimental and comparative groups, with average H-scores of 106.48 ± 35.31 and 104.30 ± 47.82, respectively (Supplementary Fig. S16).

(4) Necroptosis

Necroptosis was marked by the MLKL antibody, which was expressed moderately in both the experimental and comparative groups, with average H-scores of 156.71 ± 12.82 and 158.32 ± 26.09, respectively (Supplementary Fig. S16).

(5) Autophagy

Autophagy was marked by the LC3 antibody. Its expression ranged from low to moderate in both the experimental and comparative groups, with average H-scores of 119.44 ± 23.71 and 124.90 ± 33.48, respectively (Supplementary Fig. S16).

(6) Angiogenesis

HIF was expressed at relatively low levels in both the experimental and comparative groups, with average H-scores of 15.69 ± 20.49 and 13.52 ± 20.49, respectively. On the other hand, IL-8 was not expressed in the experimental group, with an average H-score of 8.03 ± 9.75, and its expression in the comparative group was low, with an average H-score of 57.67 ± 58.13 (Supplementary Fig. S16).

(7) Epithelial mesenchymal transition

The epithelial mesenchymal transition (EMT) was marked by E-cadherin, which showed low to moderate expression in both groups, with average H-scores of 78.12 ± 30.52 and 71.61 ± 38.79, respectively. Meanwhile, TGFβ showed low expression in both groups, with an average H-score of 44.23 ± 26.86 for the experimental group and 32.47 ± 17.98 for the comparative group, and one sample, A-1, showing negative expression. αSMA was expressed at relatively low levels in both groups, with average H-scores of 47.32 ± 41.45 and 64.00 ± 49.49 in the experimental and comparative groups, respectively (Supplementary Fig. S16).

(b) Comparison within the experimental group

The H-scores of the IHC analysis are depicted in Supplementary Fig. S17.

1. UPR

A non-significant difference and increases were observed in ATF6 and PERK. On the other hand, the expression of XBP1 was found to be lowest in recurrent tissue within 3 months and highest in recurrent tissue later than 3 months, with a significance difference between those two intervals (*p* < 0.05).

(2) Inflammatory signaling

A non-significant difference and decreases were observed in NF-κB, with the lowest average H-score found in recurrent tissue later than 3 months.

(3) Apoptosis

A non-significant difference and increases were observed in GADD153, with the highest average H-score found in recurrent tissue later than 3 months.

(4) Necroptosis

A non-significant difference and decreases were observed in MLKL, but it almost plateaued in recurrent tissue later than 3 months.

(5) Autophagy

The highest average H-score for LC3 was found in the first occurrence, and it decreased and reached a plateau in the recurrent tissue.

(6) Epithelial mesenchymal transition

The average H-score for TGFβ was highest in recurrent tissue within 3 months and lowest in recurrent tissue later than 3 months. On the other hand, αSMA and E-cadherin showed non-significant increases in their average H-scores during that time.

## Discussion

Our investigation, like prior studies^11,40,41^, identified abundant macrophages and fibroblast proliferation in FBG tissue, suggesting attempts to eliminate implanted materials. In contrast to previous studies^42–44^ that describe histological features of peri-implantitis with abundant inflammatory cells, our peri-implantitis specimens exhibited diminished inflammatory cell abundance, with lymphocytes and dendritic cells predominating, hinting at a transition beyond acute inflammation.

While microscopic features of peri-implant hyperplastic tissue are largely unexplored, our findings revealed pseudo-epitheliomatous hyperplasia within the epithelial layer, likely due to prolonged inflammation^45^. We observed a variety of inflammatory cells, including neutrophils, plasma cells, macrophages, and lymphocytes, along with signs of active keratinization and fibrosis^7^. Of note, in patient 3, FFF-derived skin during mandible reconstruction demonstrated metaplasia, showcasing the adaptability of epithelial tissues and flexibility of keratinocytes during regeneration, possibly influenced by environmental stress and local microenvironmental signals^46^.

We conducted the inaugural quantitative IHC analysis of peri-implant hyperplastic tissue using QuPath^®^, an open-source digital pathology image analysis program^47^. QuPath^®^ has been extensively used in diverse pathological investigations, elucidating complex immunopathological processes^48–58^, affirming its value in pathology research. The UPR involves three main signaling branches mediated by transmembrane proteins: inositol-requiring enzyme 1 (IRE1), PERK, and ATF6^59,60^. Our investigation detected the expression of all three UPR proteins in various cell types across comparative and experimental groups, rejecting the null hypothesis. The experimental group exhibited a significantly higher H-score for PERK, indicating its potential role in peri-implant hyperplasia tissue pathology, though further studies are warranted to definitively ascertain the primary pathway contributing to ERS due to methodological limitations. During prolonged ERS, GRP78, a chaperone protein, dissociates from UPR-initiating molecules, activating UPR sensors. Specifically, IRE1 can directly interact with NF-κB, promoting its activation and downstream inflammatory signaling^61^. The IκB kinase/NF-κB signaling pathway, integral to the pathogenesis of inflammatory diseases, exhibits heightened activation during chronic inflammation and contributes to tissue damage, particularly in epithelial tissues^62^. In the development of periodontitis and oral squamous cell carcinoma, NF-κB activation under ERS was evidenced^32^. A previous study showed that NF-κB was upregulated in peri-implantitis and correlated positively with many bacterial virulence factors^63^. In our study, the comparative group showed significantly higher H-scores for NF-κB than the experimental group, possibly due to differences in the microbiome affecting NF-κB expression, considering the FFF skin paddle’s retention of skin structure and integrity^64^. Therefore, these differences lead to the rejection of the null hypothesis.

The interaction between keratinocytes and fibroblasts is crucial for skin integrity. Dysregulated signaling during tissue injury can impair wound healing or lead to fibroproliferative disorders^65^. Keratinocytes stimulate fibroblasts via interleukin 1 production, inducing growth factors and metalloproteinases. Fibroblasts reciprocally modulate keratinocyte viability, proliferation, and differentiation. Keratinocyte survival is enhanced in the presence of fibroblasts, with reduced apoptosis and altered expression of B-cell lymphoma 2 (Bcl2) and p53^66^. Bcl2 inhibits the activation of pro-apoptotic proteins and prevents the release of cytochrome c from mitochondria, a key step in the apoptotic pathway^67^. ERS disrupts the balance between pro- and anti-apoptotic factors, influencing Bcl2 expression and CHOP/GADD153-mediated apoptosis^68,69^. Bachar-Wikstrom et al.^70^ demonstrated that increased ERS markers impair keratinocyte and fibroblast migration, as observed in our study.

The programmed cell death apoptosis is considered to prevent inflammation and unregulated and accidental cell death, whereas necroptosis is considered to induce inflammation through the release of cellular contents^71^. Necroptosis, mediated by MLKL, triggers the release of damage-associated molecular patterns (DAMPs), recruiting immune cells and inducing inflammation^72^. High MLKL expression in psoriatic skin contributes to epidermal damage, as observed in both human and animal studies^73^. These findings align with our ultrastructural observations, particularly regarding epidermal damage and MLKL expression.

During infection or injury, tissues often experience hypoxia and inflammation due to fluid accumulation and immune cell recruitment, reducing oxygen supply^74^. Crosstalk between ERS and hypoxia has been discussed^75–77^. HIF-1, a transcription factor responding to low oxygen levels, aids cell survival in hypoxic conditions and promotes expression of pro-angiogenic factors like VEGF during immune responses^78^. Our study observed low HIF-1α expression in both groups, suggesting potential induction of ERS by hypoxia. Further analyses are needed to elucidate HIF-1α’s role. While IL-8 may not primarily drive angiogenesis in our experimental group, it could contribute during oral tissue inflammation episodes like FBG^78^.

We observed low positive EMT marker expression in both groups. Type-2 EMT, notably αSMA and E-cadherin, contributes to inflammation, wound healing, and fibrosis by generating myofibroblasts from epithelial cells^79,80^. Histological differences and changes in antibody expression were noted between initial occurrences, recurrences within and beyond 3 months. Prior research on various tumors highlighted histological transformations and protein expression differences in recurrent lesions compared to primary ones^81–86^. We observed dynamic histological changes in peri-implant hyperplasia, including evolving prickle cell morphology, varying inflammatory cell composition, and changes in fibroblast shape with collagen deposition. One recurrence beyond 3 months showed a hair shaft lacking structure, indicating progressive histological transformations.

During wound healing, phases persist for up to 3 months post-injury, with scar strength reaching approximately 80% of unwounded skin, aided by fibrocytes facilitating fibroblast population and collagen synthesis^87–90^. Concurrent activation of UPR, apoptosis, and minimal suppression of autophagy in response to ERS correlates with elevated αSMA levels, indicating myofibroblast activation and potential fibrotic progression. Increased E-cadherin expression signifies establishment of an epithelial barrier during scar tissue maturation. In malignancy, elevated αSMA and reduced E-cadherin expression mark the EMT^91^, while pseudo-epitheliomatous hyperplasia typically maintains E-cadherin levels, making E-cadherin as a differentiating marker between oral squamous cell carcinoma and pseudo-epitheliomatous hyperplasia^92,93^. Our study emphasizes the noncancerous nature of peri-implant hyperplasia tissue.

A non-significant decline in TGFβ, coupled with compromised autophagy denoted by LC3, indicates dynamic cellular responses to ERS and fibrotic cues. TGFβ plays a crucial role in wound repair, particularly in fibrosis, stimulating fibroblast proliferation and collagen synthesis^94^. Its activity peaks during proliferation and early fibrotic stages but may decrease later^95^. This reflects cellular adaptation to stress-induced fibrotic changes, with implications for tissue repair and remodeling. The non-significant downregulation of MLKL, NF-κB, and LC3, alongside UPR activation, suggests cellular prioritization of survival over programmed cell death and inflammation, highlighting adaptive strategies in peri-implant hyperplastic tissue.

Our study found that peri-implant hyperplasia in FFF-reconstructed jaws, peri-implantitis, and FBG tissues exposed to a metal reconstruction plate resulted from cellular metabolic imbalance inducing ERS. Despite similarities in samples from both groups obtained from tissues surrounding titanium-based materials, studies suggest titanium and its alloys can cause inflammation and allergic reactions due to nanoparticle and ion deposition from corrosion and wear^96,97^. Titanium also induces ERS in cells, disrupting mitochondria-associated ER membranes and calcium ion balance, thereby increasing autophagy^98^. Although our study provided limited energy dispersive X-ray data indicating the presence of titanium and its alloys in hyperplastic tissue, it is plausible that these microparticles contributed to the observed tissue formation.

The association of ERS and oral pathology, including peri-implant hyperplastic tissue, peri-implantitis, and FBG tissues, involves diverse cellular signaling pathways and external factors. This study provides the first comprehensive ultrastructural and IHC analyses of peri-implant hyperplastic tissue in FFF reconstruction. Findings indicate two distinctive histological features: pseudo-epitheliomatous hyperplasia and reticular dermis fibrosis. Electron microscopy revealed evidence of ERS within cells, potentially explaining abnormalities in keratinocyte and fibroblast morphology, migration, angiogenesis, inflammatory responses, and programmed cell death (Supplementary Fig. S18).

Metal reconstruction plates trigger microbial activity-associated inflammation and subsequent ERS within peri-implant tissues. Exploring alternative materials like polyetheretherketone (PEEK) or zirconia-based materials may attenuate the inflammatory cascade, considering their low plaque affinity compared to metals^99–101^. Addressing microgaps between implant fixtures and abutments is crucial, as they serve as breeding grounds for microbial colonization and may exacerbate inflammation and ERS through galvanic currents^102,103^. Minimizing or eliminating such microgaps, adopting one-body implants, or refining abutment designs to ensure seamless integration with the implant fixture could reduce the inflammatory burden on peri-implant tissues and mitigate ERS-induced pathological changes. Exploring these interventions could bridge the gap between mechanistic insights into ERS pathology and clinically applicable strategies for preserving peri-implant tissue homeostasis and improving implant success rates.

### Supplementary Information

Supplementary Information.

## Data Availability

The datasets generated during and/or analyzed by the authors during this study are available from the corresponding author on reasonable request.
