# Supplementary material for: Ultrastructural and immunohistochemical evaluation of hyperplastic soft tissues surrounding dental implants in fibular jaws
Source: Sci Rep. 2024 May 10;14:10717. doi: 10.1038/s41598-024-60474-z (PMC11087521; doi:10.1038/s41598-024-60474-z)
Supplement: Supplementary file 1 — Supplementary Information. [file 41598_2024_60474_MOESM1_ESM.zip › S2 - Supplementary Table.docx]

**Supplementary Table S2.** Create thresholder and positive cell detection parameters in Qupath

| Create thresholder | | |
| --- | --- | --- |
| Resolution | | Extremely low (16.19 μm/px) |
| Channels | | Average channels |
| Prefilter | | Gaussian |
| Smoothing sigma | | 2 |
| Threshold | | 215 |
| Above threshold | | Unclassified |
| Below threshold | | Region |
| Region | | Everywhere |
| Classifier name | | Tissue detection |
| Positive cell detection | | |
| Setup parameters | Detection image | Optical density sum |
|  | Requested pixel size | 0.5 μm |
| Nucleus parameters | Background radius | 8 μm |
|  | Use opening by reconstruction | True |
|  | Median filter radius | 0 μm |
|  | Sigma | 1.5 μm |
|  | Minimum area | 10 μm^2 |
|  | Maximum area | 400 μm^2 |
| Intensity parameters | Threshold | 0.075 |
|  | Max background intensity | 2 |
|  | Split by shape | True |
|  | Exclude DAB (membrane staining) | False |
| Cell parameters | Cell expansion | 5 μm |
|  | Include cell nucleus | True |
| General parameters | Smooth boundaries | True |
|  | Make measurements | True |
| Intensity threshold parameters | Score compartment | Cell: DAB OD mean |
|  | Threshold 1+ | 0.075 |
|  | Threshold 2+ | 0.15 |
|  | Threshold 3+ | 0.225 |
|  | Single threshold | False |
