# Supplementary material for: Ultrastructural and immunohistochemical evaluation of hyperplastic soft tissues surrounding dental implants in fibular jaws
Source: Sci Rep. 2024 May 10;14:10717. doi: 10.1038/s41598-024-60474-z (PMC11087521; doi:10.1038/s41598-024-60474-z)
Supplement: Supplementary file 1 — Supplementary Information. [file 41598_2024_60474_MOESM1_ESM.zip › S4 - Supplementary Table.docx]

**Supplementary Table S4.** Clinical data of patients in comparative group

| Patient | PMH | Age/Sex | Diagnosis | Treatment | Affected jaw | Subjective symptoms | Objective findings |
| --- | --- | --- | --- | --- | --- | --- | --- |
| C-1 | Asthma (Under medication for 10 years) | 67/F | Chronic periimplantitis | #13i, #14i, #15i removal, mass excision, bone graft | Maxilla | Pain and swelling on the right upper jaw on the implant site/ | Positive percussion on #14i, #15i, and positive palpation on #15i |
| C-2 | Stomach cancer | 53/M | Chronic periimplantitis | #33i curettage and granulation tissue removal, #34i installation | Mandible | Discomfort on left mandible implant site | #33i inflamed tissue on labial and lingual side, BOP (+) |
| C-3 | Osteoporosis | 58/F | Chronic periimplantitis | #45i, #46i curettage, detoxification, minocycline application | Mandible | Non-specific | #45i, #46i marginal bone loss |
| C-4 | Osteoporosis (Bisphosphonate consumption for 20 years)  Saucerization on right mandible due to MRONJ followed by reconstruction using R-plate | 80/F | MRONJ | Granulation tissue curettage + dexamethasone injection | Mandible | Pain on right mandible | Granulation tissue surrounding exposed R-plate in right mandible |

*BOP, bleeding on probe; MRONJ, medication related osteonecrosis of jaws; PMH, patient medical history; R-plate, reconstruction plate.
