# Supplementary material for: Ultrastructural and immunohistochemical evaluation of hyperplastic soft tissues surrounding dental implants in fibular jaws
Source: Sci Rep. 2024 May 10;14:10717. doi: 10.1038/s41598-024-60474-z (PMC11087521; doi:10.1038/s41598-024-60474-z)
Supplement: Supplementary file 1 — Supplementary Information. [file 41598_2024_60474_MOESM1_ESM.zip › S5 - Supplementary Table.docx]

**Supplementary Table S5.** Clinical data of four patients in experimental group

| Patient | PMH | Age/sex | Diagnosis | Treatment | Affected Jaw | Implant location | Implant size | Prosthesis type |
| --- | --- | --- | --- | --- | --- | --- | --- | --- |
| 1 | Non-specific | 20/M | Fibroblastoma | Partial maxillectomy, delayed reconstruction with FFF | Maxilla | 22 | ITI^#^ 4.1 x 8.0 mm | Bridge crown |
|  |  |  |  |  |  | 23 |  |  |
| 2 | Non-specific | 32/M | Ameloblastoma | Mandibulectomy, reconstruction with FFF | Mandible | 32 | Stella^@^ 4.0 x 10.0 mm | Bar-retained overdenture |
|  |  |  |  |  |  | 35 |  |  |
|  |  |  |  |  |  | 42 |  |  |
|  |  |  |  |  |  | 45 |  |  |
| 3 | Mental illness | 34/F | Ameloblastoma | Partial mandibulectomy, reconcstruction with FFF | Mandible | 32 | ITI^#^ SP 4.1 x 8.0 mm | Bar-retained overdenture |
|  |  |  |  |  |  | 34 |  |  |
|  |  |  |  |  |  | 42 |  |  |
|  |  |  |  |  |  | 44 |  |  |
| 4 | Tongue cancer and radiotherapy | 46/F | Osteoradionecrosis | Partial mandibulectomy, reconstruction with R-plate, R-plate removal, reconstruction with FFF | Mandible | 32 | Luna^®^ 4.0 x 8.5 | Bar-retained overdenture |
|  |  |  |  |  |  | 34 | Luna^®^ 4.0 x 8.5 |  |
|  |  |  |  |  |  | 42 | Luna^®^ 4.5 x 8.5 |  |
|  |  |  |  |  |  | 44 | Luna^®^ 4.0 x 8.5 |  |

^®^Luna (Shinhung Implant System, Seoul, Korea), ^@^Stella (Shinhung Implant System, Seoul, Korea), ^#^ITI SP: standard plus (Institut Straumann AG, Basel, Switzerland).

FFF, fibula free flap; PMH, patient medical history; R-plate, reconstruction plate
