# Supplementary material for: Ultrastructural and immunohistochemical evaluation of hyperplastic soft tissues surrounding dental implants in fibular jaws
Source: Sci Rep. 2024 May 10;14:10717. doi: 10.1038/s41598-024-60474-z (PMC11087521; doi:10.1038/s41598-024-60474-z)
Supplement: Supplementary file 1 — Supplementary Information. [file 41598_2024_60474_MOESM1_ESM.zip › S6 - Supplementary Table.docx]

**Supplementary Table S6.** Distribution of experimental specimens into subgroups

| **Subgroup A** | **B** | **Ce** | **D** | **E** | **F** | **G** | **H** |
| --- | --- | --- | --- | --- | --- | --- | --- |
| A-1 | B-2 | Ce-1 | D-1 | E-1 | F-1 | G-4 | H-4 |
| A-2 |  |  | D-2 | E-3 | F-3-1 |  |  |
|  |  |  | D-3 |  | F-3-2 |  |  |

*-1, -2, -3, and -4 refer to Patient 1, Patient 2, Patient 3, and Patient 4. Additionally, the specimens were labeled according to the tissue excision interval (A-H). A; First occurrence, B; Recurrence, one-month interval excision, Ce; Recurrence, two-month interval excision, D; Recurrence, three-month interval excision, E; Recurrence, four-month interval excision, F; Recurrence, six-month interval excision, G; Recurrence, one-year interval excision, H; Recurrence, more than one-year interval excision. Example; “A-2” indicating “recurrence tissue excised at one-month interval in patient 2”, F-3-1. F-3-2; two specimens from different recurred mass at 6 months interval were retrieved from patient 3.
