# Supplementary material for: Ultrastructural and immunohistochemical evaluation of hyperplastic soft tissues surrounding dental implants in fibular jaws
Source: Sci Rep. 2024 May 10;14:10717. doi: 10.1038/s41598-024-60474-z (PMC11087521; doi:10.1038/s41598-024-60474-z)
Supplement: Supplementary file 1 — Supplementary Information. [file 41598_2024_60474_MOESM1_ESM.zip › Supplementary Figure Legends.docx]

**Supplementary Figure Legends**

**Supplementary Figure S1.** Current understanding of endoplasmic reticulum stress (ERS) and its implications for systemic diseases.

**Supplementary Figure S3.** Qupath^®^ setting used for this study: estimate stain vectors (a), create a threshold (b), detect positive cells (c), and calculate resulting H-scores (d).

**Supplementary Figure S7.** H&E findings of peri-implantitis specimens: C-1 (A1–3), C-2 (B1–3), C-3 (C1–3), and C-4 (D1–3). The epidermal layer shows thickening of the stratum spinosum with a majority of nucleated prickle cells (A2). Fibrotic tissue filled by collagen fibers, inflammatory cells (A3; sequestrum was observed marked by #, B2, C3), and abundant blood vessels (B3). Acanthosis of the stratum spinosum with an intact stratum basalis was observed (D2). In the fibrotic reticular dermis of FBG, abundant blood vessels and inflammatory cells can be seen (D1).

**Supplementary Figure S8.** Scanning electron microscope (SEM) findings from the A-1 and A-2 specimens revealed that the stratum spinosum consisted of anucleated prickle cells with inflammatory cells (arrowhead), bacteria (arrows), and keratinocytes (yellow asterisk) (a1–2). Bacteria in the middle of the stratum spinosum (a3). The formation of extracellular matrix, with new blood vessels and thick connective tissue, was evidenced (arrows) (b1). Neutrophil (b2) and monocyte (b3)**.** SEM findings of the Ce-1 specimen revealed thick keratinized epidermis (c1), stratum corneum rich in keratin granules (c2), and stratum spinosum consisting of mixed anucleated and nucleated prickle cells (c3). The formation of extracellular matrix, with new blood vessels, thick connective tissue, and abundant fibroblasts (d1–3).

**Supplementary Figure S9.** SEM findings revealed angiogenesis. The early development of blood vessels (yellow arrows) was seen to be surrounded by inflammatory cells and fibroblasts at 2500x (A1) and 5000x magnifications (A2). At 30,000x the early development of blood vessels shows the basic structure of endothelial cells without lumen (A3). B1–B3 show the development of the lumen (blue arrow) in the blood vessel surrounded by endothelial cells, with fewer inflammatory cells. A mature blood vessel containing blood cells can be observed with fewer inflammatory cells and more fibroblast cells (C1–C3). A small mature blood vessel containing lymphocytes (D1–D3). A mature blood vessel can be seen containing monocytes and other mononuclear cells (E1–E3). Abbreviations: E; endothelial cell, F; fibroblast, L; lymphocyte, M; macrophage, Mo; monocyte, N; neutrophil.

**Supplementary Figure S10.** Transmission electron microscope (TEM) findings of blood vessel structure and peripheral blood cells. Mature blood vessels with complete structures, including pericytes and smooth muscles, can be seen in A (1000x, 5 µm), B (1000x, 5 µm), and C (2000x, 2µm). In A and B, neutrophils can be seen exiting the blood vessel. At 5000x magnification, platelets with an open canalicular system (yellow arrowheads) and alpha granule structures can be seen near apoptotic bodies in the dense connective tissue (D). A cell with a multilobed nucleus and highly condensed heterochromatin at 5000x magnification (E). Lymphocyte cell with pseudopod (yellow arrowheads) at 5000x magnification (F). Mast cell at 3000x magnification (G). A clearer image of secretion granules at 5000x magnification (H). Abbreviations: α; alpha granules, AB: apoptotic bodies, CT; connective tissue, DCT; dense connective tissue, E; endothelial, F; fibroblast, N: neutrophil; P; pericyte, SG; secretion granule, SM; smooth muscle.

**Supplementary Figure S11.** SEM findings of the most abundant inflammatory cells in the reticular dermis. Macrophages (blue arrowheads) and neutrophils (yellow arrowheads) can be seen scattered around the reticular dermis (a, 5000x magnification). A 10,000x magnification view of macrophages (b). Plasma cell with prominent endoplasmic reticulum (ER) and spoke wheel nucleus (red arrowheads) and macrophages (blue arrowheads) at 10,000x magnification (c). Two plasma cells (red arrowheads) in intimate proximity and a lymphocyte–dead cell interaction (green arrowhead) at 10,000x magnification (d). Detail of a plasma cell showing large amounts of rough ER and a spoke-wheel nucleus (e, 30,000x magnification). Binucleated plasma cell as a result of cell fusion (f, 30,000x magnification). TEM findings of inflammatory cells and the death of a cell. Dendritic cell with pseudopod (blue arrowheads) in the connective tissue (yellow arrow indicates collagen thread) (g, 3000x magnification). Macrophage structure (h, 5000x magnification). Two lymphocytes interact with a macrophage and apoptotic bodies (i, 3000x magnification). At 5000x magnification, a close interaction between a lymphocyte and a macrophage (yellow arrowheads) can be seen (j). A cell undergoing apoptosis, with nuclear fragmentation in the cytoplasm bleb (k, 5000x magnification). A cell undergoing oncosis: cytoplasm blebs (blue arrowheads), dilated mitochondria, karyorrhexis of the nucleus, and disruption of the membrane (black arrows) can be seen (l, 10,000x magnification). Abbreviations: AB; apoptotic bodies, M: mitochondria.

**Supplementary Figure S12.** SEM findings show metaplasia of prickle cells into cuboidal cells in specimen E-3 (A1–2). Acanthosis with metaplasia was observed in F-3-1 (B1–2), and abundant inflammatory cells and programmed cell death were observed (C1–2). Bacterial invasion was observed in specimen H-4 (C3–4).

**Supplementary Figure S13.** The G-4 specimen showed that metaplasia of the prickle cells in the epidermal layer was characterized by less density, and the high-density stratum spinosum anastomosed into the reticulum dermal layer (A1). Abundant lymphocytes and dendritic cells were observed between the epidermal and reticulum dermal layers (A2–4). Cells undergoing degenerative changes were also observed (A5).

**Supplementary Figure S14.** Zebra bodies (A1) and a myelin figure (A2) were observed in specimen C-1, marking necrosis and degenerative changes. Fibroblasts with degenerative changes of the organelles (B1) and abundant inflammatory cells (B2). Degenerative changes or programmed cell death (C1, C2).

**Supplementary Figure S15.** SEM examination of FBG. The intact stratum basalis can be seen (a, b). Abundant macrophages and dendritic cells can be seen in the reticular dermis (c1). Plasma cells and neutrophils were also seen (c2). A large blood vessel with an invasion of bacteria inside (d1, d2). Phagocytosis of a dead fibroblast (e1); at higher magnification, a fragmented and dilated ER was seen (e2). Fibroblasts surrounded by inflammatory multinucleated cells were observed (f1), and at high magnification, a dilated and fragmented ER was seen (f2).

**Supplementary Figure S16.** The IHC for programmed death cell, angiogenesis, EMT, and inflammatory signaling.

**Supplementary Figure S17.** Statistical analysis was performed with one-way ANOVA and post-hoc Tukey testing. The expression comparison of ATF6 (a), PERK (b), and XBP1 showed significant differences between recurrences within 3 months and later than 3 months (c), NF-κB (d), GADD153 (e), MLKL (f), LC3 (g), TGFβ (g), αSMA (h), and E-cadherin (i). ^#^aa; first occurrence, bb; recurrence within 3 months, cc; recurrence later than 3 months.

*αSMA, α-smooth muscle actin; ATF6, activating transcription factor 6; CHOP/GADD153, CCAAT-enhancer-binding protein homologous protein/growth arrest-and DNA damage-inducible gene 153; E-cadherin, epithelial cadherin; LC3, microtubule-associated protein 1 light chain 3; MLKL. mixed lineage kinase domain-like protein; NF-κB, nuclear factor kappa–light-chain-enhancer of activated B cells; PERK, protein kinase RNA activated (PKR-like ER kinase); TGFβ, transforming growth factor beta; XBP1, X-box-binding protein 1.

**Supplementary Figure S18.** Schematic drawing of a possible mechanism for peri-implant hyperplastic tissue in fibular jaw formation.
