# Supplementary figures and images for: Ultrastructural and immunohistochemical evaluation of hyperplastic soft tissues surrounding dental implants in fibular jaws
Source: Sci Rep. 2024 May 10;14:10717. doi: 10.1038/s41598-024-60474-z (PMC11087521; doi:10.1038/s41598-024-60474-z)

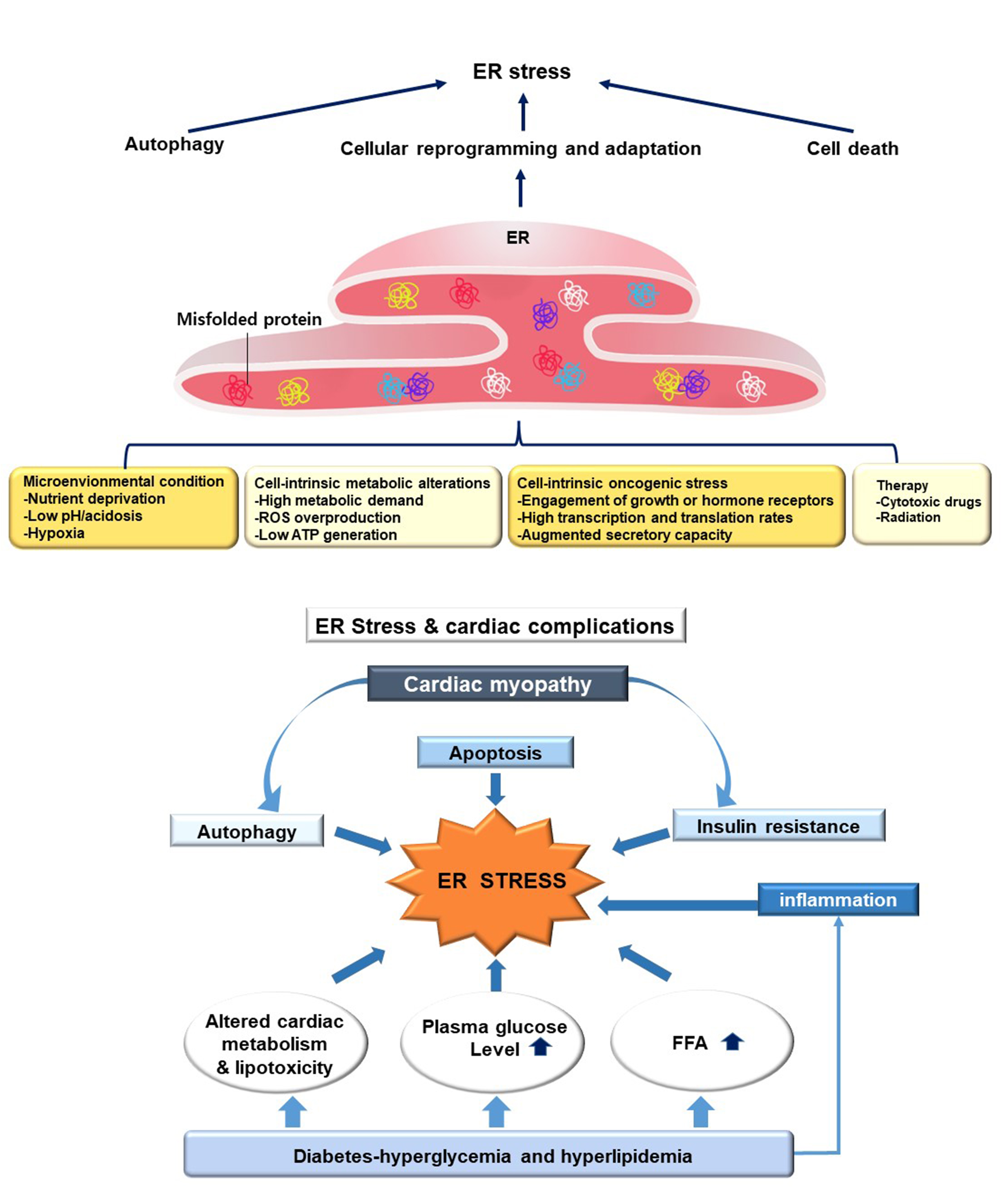

Supplement: Supplementary file 1 — Supplementary Information. [file 41598_2024_60474_MOESM1_ESM.zip › S1 - Supplementary Figure.jpg]

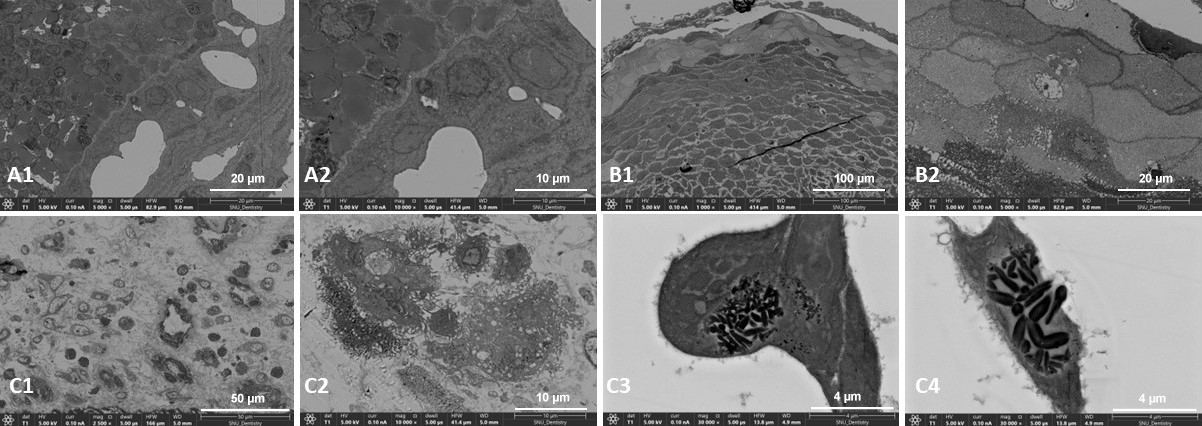

Supplement: Supplementary file 1 — Supplementary Information. [file 41598_2024_60474_MOESM1_ESM.zip › S12 - Supplementary FIgure.jpg]

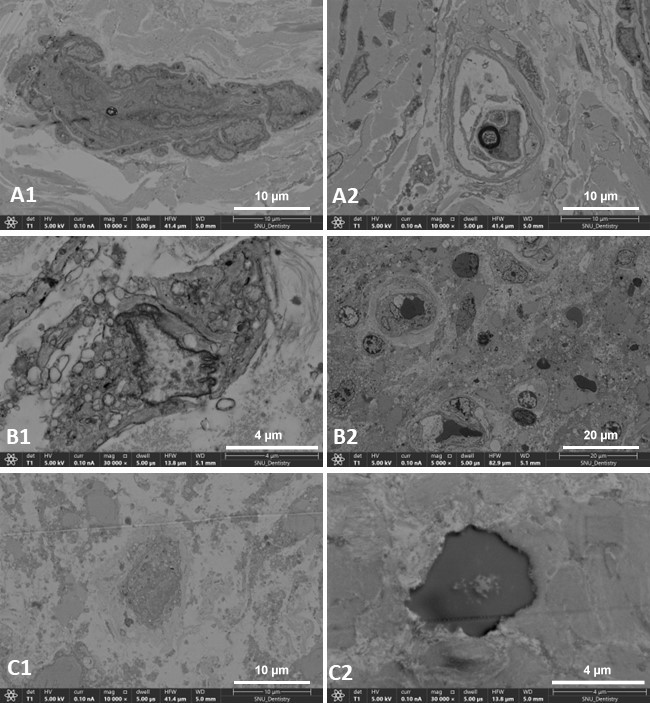

Supplement: Supplementary file 1 — Supplementary Information. [file 41598_2024_60474_MOESM1_ESM.zip › S14 - Supplementary FIgure.jpg]

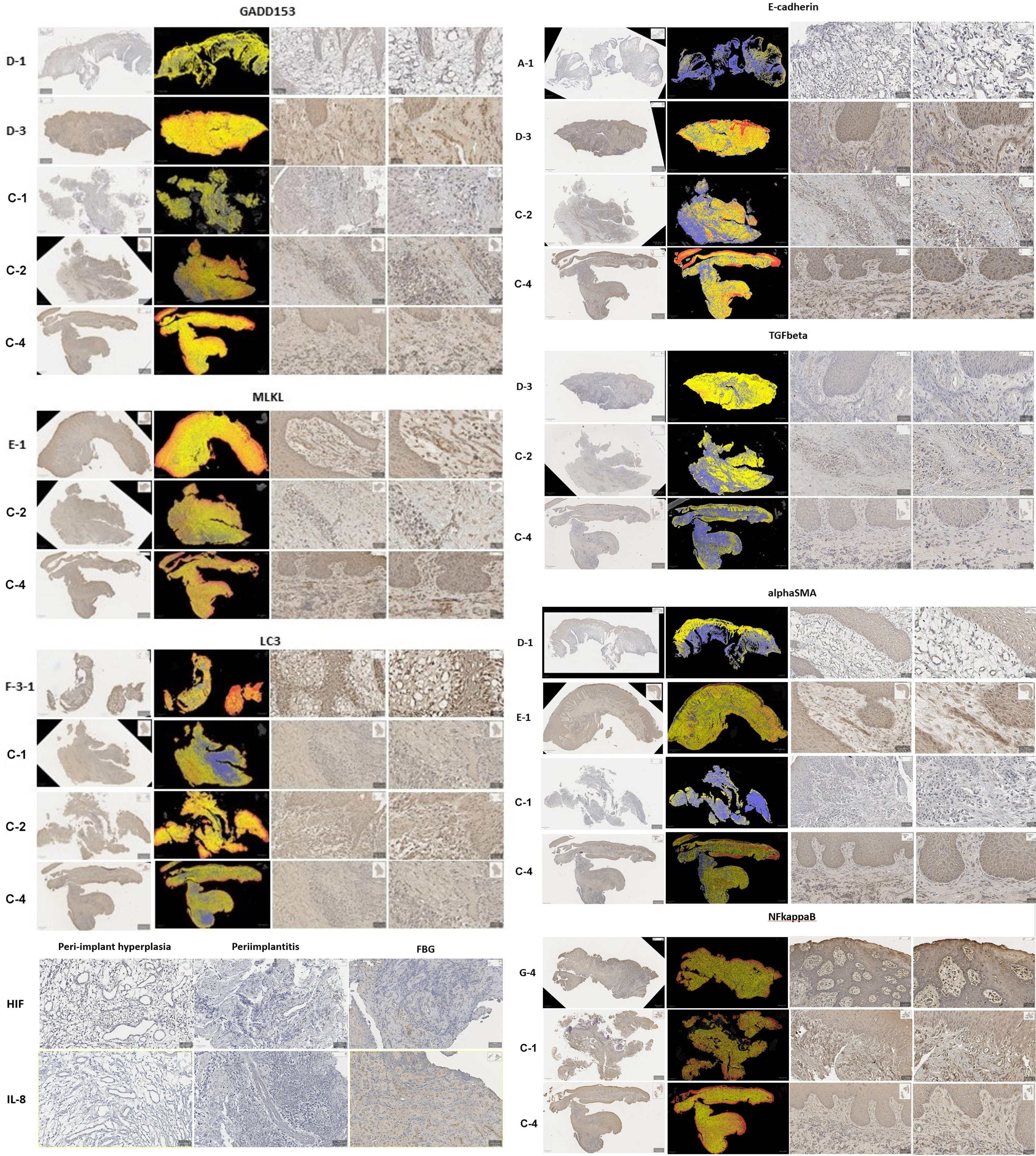

Supplement: Supplementary file 1 — Supplementary Information. [file 41598_2024_60474_MOESM1_ESM.zip › S16 - Supplementary Figure.jpg]

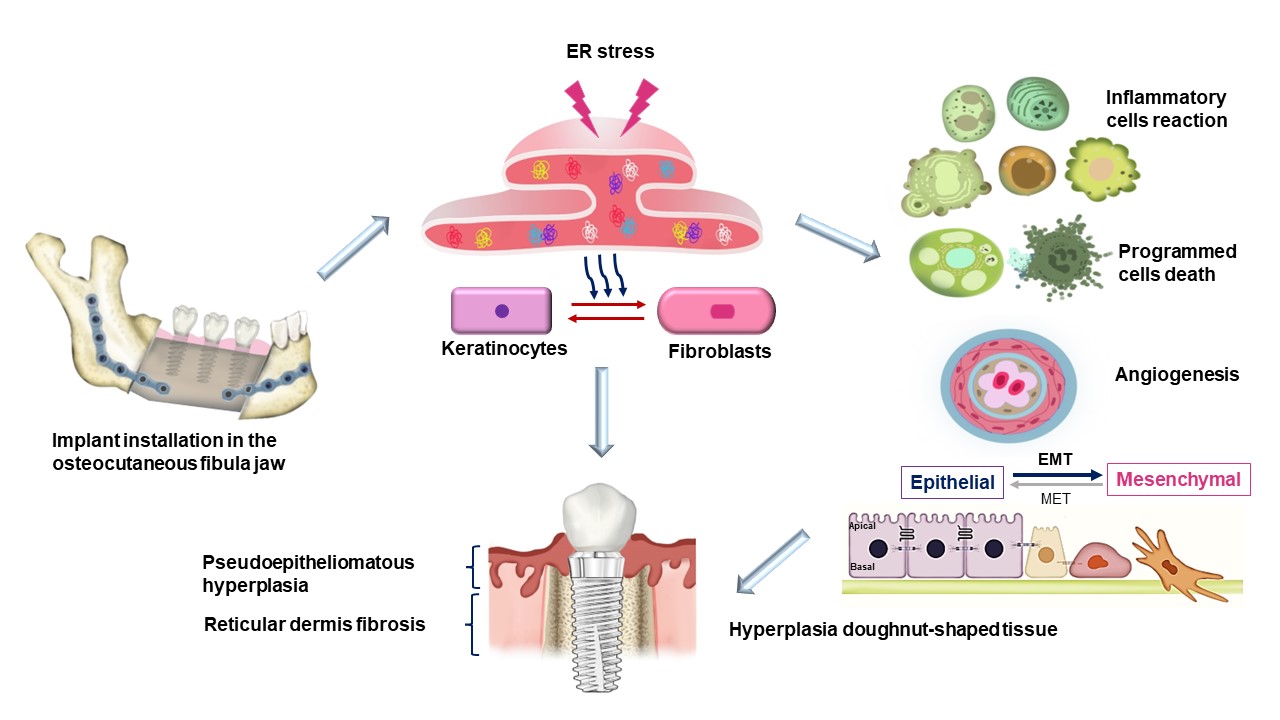

Supplement: Supplementary file 1 — Supplementary Information. [file 41598_2024_60474_MOESM1_ESM.zip › S18 - Supplementary FIgure.jpg]

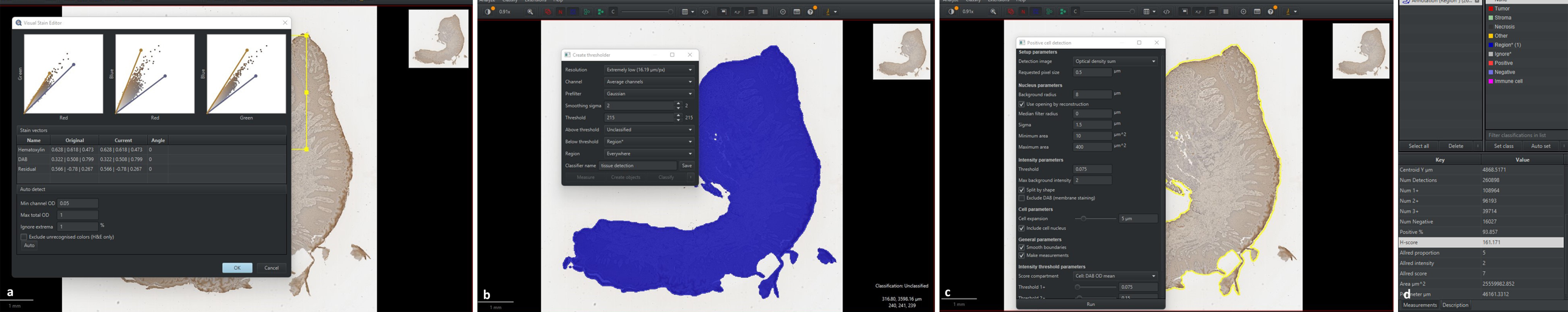

Supplement: Supplementary file 1 — Supplementary Information. [file 41598_2024_60474_MOESM1_ESM.zip › S3 - Supplementary Figure.jpg]

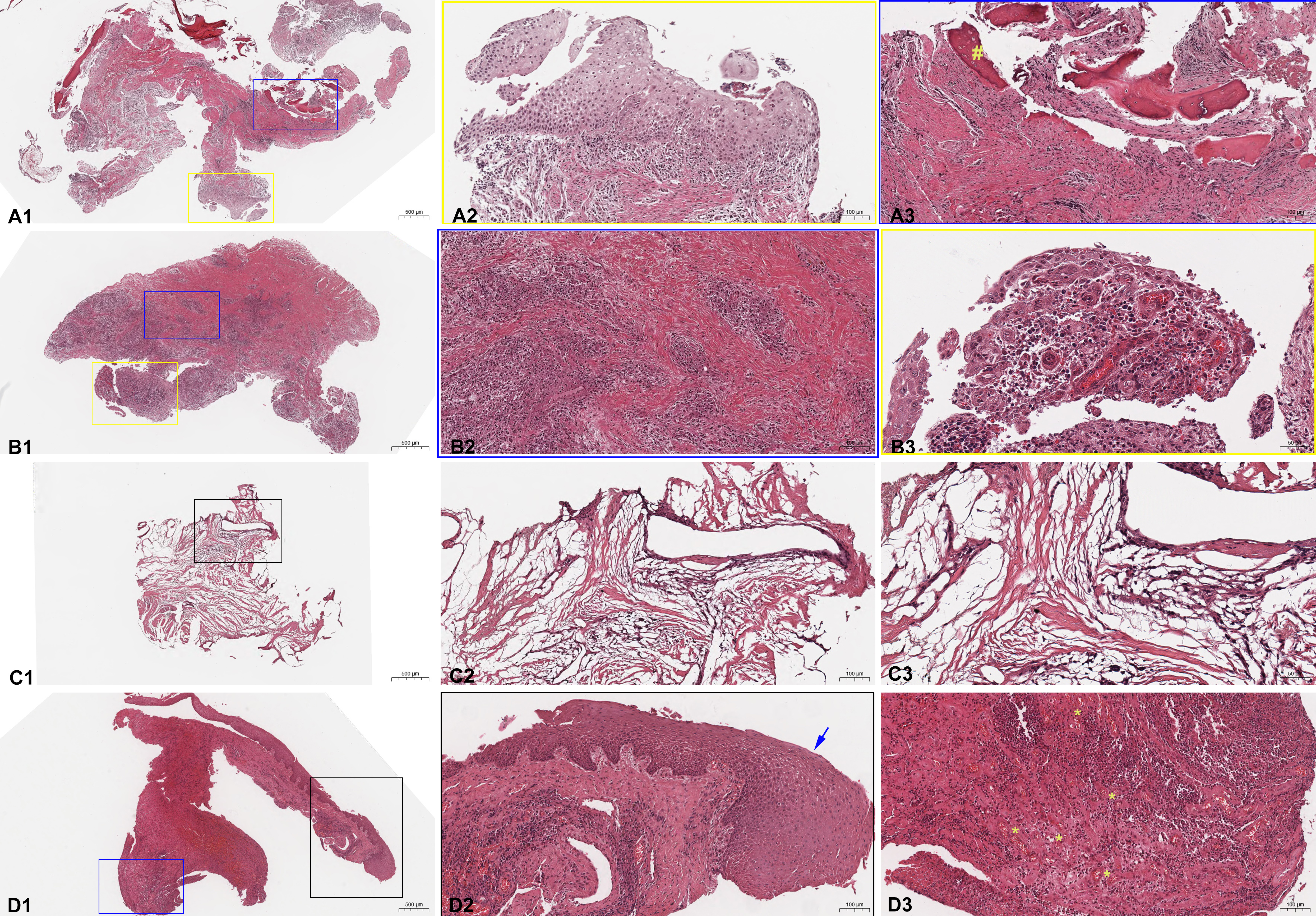

Supplement: Supplementary file 1 — Supplementary Information. [file 41598_2024_60474_MOESM1_ESM.zip › S7 - Supplementary Figure.jpg]
